# Correction: Improving the Robustness and Clinical Applicability of Automatic Respiratory Sound Classification Using Deep Learning–Based Audio Enhancement: Algorithm Development and Validation

**DOI:** 10.2196/76150

**Published:** 2025-04-29

**Authors:** Jing-Tong Tzeng, Jeng-Lin Li, Huan-Yu Chen, Chun-Hsiang Huang, Chi-Hsin Chen, Cheng-Yi Fan, Edward Pei-Chuan Huang, Chi-Chun Lee

**Affiliations:** 1 College of Semiconductor Research National Tsing Hua University Hsinchu Taiwan; 2 Department of Electrical Engineering National Tsing Hua University Hsinchu Taiwan; 3 Department of Emergency Medicine National Taiwan University Hospital Hsin-Chu Branch Hsinchu Taiwan; 4 Department of Emergency Medicine National Taiwan University Hospital Taipei Taiwan

In “Improving the Robustness and Clinical Applicability of Automatic Respiratory Sound Classification Using Deep Learning–Based Audio Enhancement: Algorithm Development and Validation” (JMIR AI 2025;4:e67239) the authors made four corrections.

The fourth author’s name has been corrected from:

Chu-Hsiang Huang

to:

Chun-Hsiang Huang

Additionally, the affiliation of authors Chun-Hsiang Huang, Chi-Hsin Chen, and Cheng-Yi Fan has been updated from:

3 Department of Emergency Medicine, National Taiwan University Hsin-Chu Hospital, Hsinchu, Taiwan

to:

*3 Department of Emergency Medicine, National Taiwan University Hospital Hsin-Chu Branch, Hsinchu, Taiwan*

Edward Pei-Chuan Huang’s affiliation has also been updated from:

3 Department of Emergency Medicine, National Taiwan University Hsin-Chu Hospital, Hsinchu, Taiwan

to:

3 Department of Emergency Medicine, National Taiwan University Hospital Hsin-Chu Branch, Hsinchu, Taiwan

4 Department of Emergency Medicine, National Taiwan University Hospital, Taipei, Taiwan

Similarly, Chi-Chun Lee’s affiliation has been updated from:

2 Department of Electrical Engineering, National Tsing Hua University, Hsinchu, Taiwan

to:

1 College of Semiconductor Research, National Tsing Hua University, Hsinchu, Taiwan

2 Department of Electrical Engineering, National Tsing Hua University, Hsinchu, Taiwan

The correction will appear in the online version of the paper on the JMIR Publications website, together with the publication of this correction notice. Because this was made after submission to PubMed, PubMed Central, and other full-text repositories, the corrected article has also been resubmitted to those repositories.

