# Supplementary material for: Improving the Robustness and Clinical Applicability of Automatic Respiratory Sound Classification Using Deep Learning–Based Audio Enhancement: Algorithm Development and Validation
Source: JMIR AI. 2025 Mar 13;4:e67239. doi: 10.2196/67239 (PMC11950698; doi:10.2196/67239)
Supplement: Multimedia Appendix 1 [file ai_v4i1e67239_app1.docx]

**Appendix 1. Technical setup**

| **Hardware** |  |
| --- | --- |
| CPU | AMD EPYC 7742 64-Core |
| GPU | NVIDIA A100 80G |
| RAM | 512 GB |
| **Library** |  |
| PyTorch | 1.12.1 |
| Torchaudio | 0.12.1 |
| NumPy | 1.23.1 |
| pandas | 1.5.1 |
| Librosa | 0.9.2 |
