# Supplementary material for: Improving the Robustness and Clinical Applicability of Automatic Respiratory Sound Classification Using Deep Learning–Based Audio Enhancement: Algorithm Development and Validation
Source: JMIR AI. 2025 Mar 13;4:e67239. doi: 10.2196/67239 (PMC11950698; doi:10.2196/67239)
Supplement: Multimedia Appendix 2 [file ai_v4i1e67239_app2.docx]

**Appendix 2. Hyperparameter Settings for Model Training**

| **Model** | **Hypermeters** | **Value** |
| --- | --- | --- |
| Wave-U-Net | Batch size | 4 |
|  | Learning rate (from scratch) | 10^-4^ |
|  | Learning rate (with pre-trained weight) | 10^-5^ |
|  | Epoch (from scratch) | 40 |
|  | Epoch (with pre-trained weight) | 30 |
|  | Channel size | 24 |
|  | Layers of up & down convolution | 8 |
| MANNER | Batch size | 4 |
|  | Learning rate (from scratch) | 10^-5^ |
|  | Learning rate (with pre-trained weight) | 10^-6^ |
|  | Epoch (from scratch) | 10 |
|  | Epoch (with pre-trained weight) | 10 |
|  | Channel size | 60 |
|  | Layers of up & down convolution | 4 |
| PHASEN | Batch size | 4 |
|  | Learning rate (from scratch) | 5⨯10^-4^ |
|  | Learning rate (with pre-trained weight) | 5⨯10^-5^ |
|  | Epoch (from scratch) | 30 |
|  | Epoch (with pre-trained weight) | 20 |
|  | Window size | 25ms |
|  | Hop size | 10ms |
| CMGAN | Batch size | 4 |
|  | Learning rate (from scratch) | 5⨯10^-4^ |
|  | Learning rate (with pre-trained weight) | 5⨯10^-5^ |
|  | Epoch (from scratch) | 30 |
|  | Epoch (with pre-trained weight) | 20 |
|  | Window size | 25ms |
|  | Hop size | 6.25ms |
| CNN14 | Batch size | 32 |
|  | Learning rate | 10^-4^ |
|  | Epoch | 140 |
|  | Window size | 32ms |
|  | Hop size | 10ms |
|  | Number of mel filterbanks | 64 |
| AST | Batch size | 8 |
|  | Learning rate | 5⨯10^-5^ |
|  | Epoch | 50 |
|  | Window size | 64ms |
|  | Hop size | 32ms |
|  | Number of mel filterbanks | 128 |
| Patch-Mix | Batch size | 8 |
|  | Learning rate | 5⨯10^-5^ |
|  | Epoch | 50 |
|  | Window size | 64ms |
|  | Hop size | 32ms |
|  | Number of mel filterbanks | 128 |
